# Adiponectin gene and risk of colorectal cancer

**DOI:** 10.1038/bjc.2011.259

**Published:** 2011-08-09

**Authors:** M C Gornick, G Rennert, V Moreno, S B Gruber

**Affiliations:** 1 Department of Human Genetics, University of Michigan School of Medicine, 1524 BSRB, 109 Zina Pitcher, Ann Arbor, MI, USA; 2 Department of Community Medicine and Epidemiology, Clalit Health Service National Cancer Control Center, Carmel Medical Center and B. Rappaport Faculty of Medicine, Technion-Israel Institute of Technology, Haifa, Israel; 3 Institut Català d'Oncologia (ICO), IDIBELL, CIBER-ESP, L'Hospitalet de Llobregat, Spain; 4 Department of Internal Medicine, University of Michigan School of Medicine, 1524 BSRB, 109 Zina Pitcher, Ann Arbor, MI, USA; 5 Department of Epidemiology, University of Michigan School of Public Health, 1524 BSRB, 109 Zina Pitcher, Ann Arbor, MI, USA

**Keywords:** adiponectin, *ADIPOQ*, colorectal cancer

## Abstract

**Background:**

Genes of the adiponectin pathway are interesting candidates for colorectal cancer risk based on the potential association between colorectal cancer and obesity. However, variants of the adiponectin gene (*ADIPOQ*) have been demonstrated to be inconsistently associated with risk of colorectal cancer.

**Methods:**

The current study attempted to evaluate these findings by examining several single nucleotide polymorphisms (SNPs) that were previously genotyped as part of a genome-wide association study in the *ADIPOQ* gene. Genotyping was also performed for a previously reported risk variant, rs266729, in 1062 individuals with a diagnosis of colorectal cancer and 1062 controls matched on age, gender and ethnicity (Jewish or not Jewish) as part of a population-based case–control study in Israel.

**Results:**

No evidence was found for an association between *ADIPOQ* and risk of colorectal cancer. The single nucleotide variant previously associated with decreased risk of colorectal cancer, rs266729, revealed an adjusted odds ratio of 1.04; 95% confidence interval, 0.88–1.23.

**Conclusion:**

The SNP, rs266729, was not strongly associated with colorectal cancer in patients of Ashkenazi Jewish descent or other ethnic groups in Israel.

Decreased levels of circulating adiponectin, a hormone secreted by the adipose tissue, have been found to be directly associated with obesity and hyperinsulinemia (Vona-Davis *et al*, 2007). The combination of the association of adiponectin with insulin resistance, support for an association of obesity with risk of colorectal cancer, and a previous report which found that adiponectin levels were inversely associated with risk of colorectal cancer suggest that the adiponectin pathway may contribute to colorectal carcinogenesis (Wei *et al*, 2005; Moghaddam *et al*, 2007; Vona-Davis *et al*, 2007; Fenton *et al*, 2008; Williams *et al*, 2008). An association between genes of the adiponectin pathway and risk of colorectal cancer was recently reported in a multicenter case–control study (Kaklamani *et al*, 2008). Kaklamani *et al* identified a single nucleotide polymorphism (SNP, rs266729) in the adiponectin gene (*ADIPOQ*) that was associated with decreased risk of colorectal cancer. However, subsequent reports did not validate the findings of Kaklamani (Carvajal-Carmona *et al*, 2009; Pechlivanis *et al*, 2009). In response to this intriguing hypothesis and inconsistent data, we examined several SNPs in the *ADIPOQ* gene that were genotyped as part of an ongoing genome-wide association study (GWAS) (Gruber *et al*, 2007). We also specifically genotyped the variant rs266729 in 1062 colorectal cancer cases and 1062 matched controls.

## Materials and methods

Histopathologically confirmed cases of all incident colorectal cancer diagnosed in northern Israel between 31 March 1998 and 31 March 2004 were recruited as part of the Molecular Epidemiology of Colorectal Cancer study. Population-based controls were identified from the Clalit Health Service database and matched to cases by year of birth, gender, clinic and Jewish/Arab ethnicity (Poynter *et al*, 2005). The study was approved by all relevant IRBs in the US and Israel, and written informed consent was given by study participants. SNPs within *ADIPOQ* were analysed as part of our GWAS to test the hypothesis that SNPs in linkage disequilibrium with the published risk variants of *ADIPOQ* were associated with risk (Table 1). Stage 1 of our GWAS (Gruber *et al*, 2007) used pooled DNA of cases (*n*=500) and pooled DNA of controls (*n*=500). Standard errors used for *t*-tests of pooled allele frequencies were corrected by adding a chip-specific constant to avoid biased selection of SNPs with small standard errors (Table 2b).

Our subsequent validation analysis had 95% power to detect an odds ratio (OR) of 0.73 by individually genotyping genomic DNA from 1062 matched pairs using Taqman SNP allelic discrimination (Table 1). Less than 5% of genotypes were scored as equivocal and 1% of the sample was genotyped in duplicate with 100% concordance. Conditional logistic regression was used to calculate ORs in R (version 2.11.1, R Development Core Team, http://www.R-project.org) and SAS (version 9.1, SAS Institute Inc., Cary, NC, USA). Analyses were adjusted for ethnicity (Ashkenazi Jewish, Sephardi Jewish, Arab) and *APC* I1307K.

## Results

Our GWAS study did not identify a significant association between any of the genotyped SNPs in the *ADIPOQ* gene and risk of colorectal cancer (Table 2a). Using the dominant model to replicate Kaklamani's reported findings, SNP rs266729 revealed an adjusted OR (adjusted for age, gender, ethnicity and *APC*I1307K status) of 1.04; 95% confidence interval (95% CI), 0.88–1.23. The OR among Ashkenazi Jews only was 1.01 (95% CI 0.82–1.24). Excluding overlapping cases and controls used in both the initial pooled GWAS analysis and the individual genotyping of the subsequent validation study yielded an adjusted OR=1.12, 95% CI (0.91, 1.38). The SNP rs266729 was in Hardy–Weinberg equilibrium in the controls. The prevalence of the CC genotype differed between the current study and the report by Kaklamani (55 *vs* 51%, *P*-value=0.02478). Therefore, we compared the MECC cases to the combined control group of Kaklamani *et al* and calculated an OR=1.10, 95% CI (0.92, 1.32). Based on the current data we conclude that rs266729 in *ADIPOQ* is not associated with risk of colorectal cancer in a comparable population-based sample.[Table tbl2b]

## Conclusion

In contrast to one previous publication (Kaklamani *et al*, 2008) but consistent with two others (Carvajal-Carmona *et al*, 2009; Pechlivanis *et al*, 2009), we found no association with variants of *ADIPOQ* and risk of colorectal cancer. It should be noted that in the study by Carvajal-Carmona, genotypes at rs266729 were imputed in the CORGI cohort, but confirmed the absence of association with CRC risk. Based on the results from the current study, rs266729 was not associated with colorectal cancer in patients of Ashkenazi Jewish descent or other ethnic groups in Israel. It seems likely that the original publication represents a ‘winner's curse’, or a chance observation in an initial study, as our larger, population-based study of the same ethnic group appears to be representative of the age and sex distribution of colorectal cancer among most western populations. Together with the data from the present study, a recent study by Pechlivanis *et al* reports that it is unlikely that genetic variation in *ADIPOQ* confers risk of colorectal cancer. However, we cannot exclude a weak association particularly because some studies had shown a differential effect in populations from the United States *vs* other European populations.

## Figures and Tables

**Table 1:** Study characteristics of GWAS and validation sample

	**Cases** *	**Controls** *
*Pooled GWAS sample*		
Age (years)	69.9 (s.d.=11.7)	70.0 (s.d.=11.7)
% Ashkenazi	100%	100%
% Male	51%	50%
		
*Validation sample*		
Age (years)	70.1 (s.d.=11.5)	71.1 (s.d.=11.5)
% Ashkenazi	90%	90%
% Male	51%	52%

Abbreviation: GWAS=genome-wide association study.

^*^The participation rate was 67.5% among eligible cases and 52.1% of eligible controls. Participating cases were slightly younger (mean 70.1 years) than cases who declined (mean 70.4 years), whereas those who could not participate because they were too ill or died were significantly older (mean 78.9 years).

**Table 2a:** *ADIPOQ* allele frequencies in DNA pools of 500 Ashkenazi Jewish cases and 500 Ashkenazi Jewish controls from GWAS

**db SNP ID**	***r* ^2^**	**Allele frequency in cases**	**Allele frequency in controls**	**Allele frequency difference (delta phat)**	**Uncorrected *P*-values**	***P*-value^a^**
rs16861194	—	0.823713	0.827086	−0.00337	0.91	0.94
rs182052	0.12	0.351247	0.347727	0.00352	0.75	0.87
rs822395	NA	0.332435	0.359826	−0.02739	0.43	0.54
rs3821799	0.15	0.725486	0.727804	−0.00232	0.93	0.95
rs6773957	0.78	0.420488	0.411588	0.00890	0.66	0.77
rs1063537	0.21	0.814909	0.768118	0.04679	0.03	0.12
rs2082940	1.00	0.103103	0.133517	−0.03041	0.11	0.30

Abbreviations: GWAS=genome-wide association study; NA=non-applicable; SNP=single nucleotide polymorphism.

a *P*-values derived from corrected *t*-test using a constant to correct the allele frequency difference. *P*-values are not adjusted for multiple comparisons. Note that the highest *r*^2^ value between rs266729 and any of the SNPs included in the GWAS study is 0.74 (rs182052).

**Table 2b tbl2b:** *ADIPOQ* SNP rs266729 genotypes in the MECC study

**Genotypes**	**Controls**	**Cases**	**OR (95% CI)^a^**	***P*-value**
*Total sample*	1062	1062		
CC	588	576	1.00 (Reference)	
CG/GG	474	486	1.04 (0.88–1.23)	0.66
				
*Ashkenazi Jews*	672	741		
CC	365	400	1.00 (Reference)	
CG/GG	307	341	1.01 (0.82–1.24)	0.98
				
*Sephardi Jews*	267	191		
CC	154	103	1.00 (Reference)	
CG/GG	113	88	1.18 (0.81–1.73)	0.38
				
*Arab*	106	111		
CC	59	63	1.00 (Reference)	
CG/GG	47	48	0.91 (0.53–1.57)	0.74

Abbreviations: CI=confidence interval; OR=odds ratio; SNP=single nucleotide polymorphism.

a Adjusted for ethnicity and *APC* I1307K status.
